# Heart ventricular histology and microvasculature together with aortic histology and elastic lamellar structure: A comparison of a novel dual-purpose to a broiler chicken line

**DOI:** 10.1371/journal.pone.0214158

**Published:** 2019-03-21

**Authors:** George Harash, Kenneth C. Richardson, Zaher Alshamy, Hana Hünigen, Hafez Mohamed Hafez, Johanna Plendl, Salah Al Masri

**Affiliations:** 1 Institute of Veterinary Anatomy, Department of Veterinary Medicine, Freie Universität Berlin, Berlin, Germany; 2 College of Veterinary Medicine, School of Veterinary and Life Sciences, Murdoch University, Murdoch, Australia; 3 Institute of Poultry Diseases, Department of Veterinary Medicine, Freie Universität Berlin, Berlin, Germany; Tokat Gaziosmanpasa University, TURKEY

## Abstract

The use of dual-purpose chickens is a strategy to avoid killing one-day-old male chicks of egg laying lines. Lohmann Dual (LD) is a novel dual-purpose chicken line created by the crossbreeding of layer and broiler lines. However, many of the cardiovascular diseases of broilers are likely to be associated with intensive genetic selection for growth and feed conversion efficiency. This study aimed to compare the macroscopic and microscopic structure of the heart and the aorta of the LD chicken line with that of the broiler chicken line, Ross 308 (Ross) under typical husbandry conditions for meat production. Eighty, one-day-old male chicks of each line were housed for 5 weeks (Ross) and 9 weeks (LD). Six birds of each line were sampled weekly. Heart mass, thickness of ventricular walls, cardiomyocyte size and blood capillary density as well as aortic diameter and thickness, number of elastic lamellae and elastic fiber percentage in the aortic wall were determined. The growth patterns of the heart were the same in the two lines. Although LD chickens had a lower absolute heart mass than that of Ross chickens, the relative heart mass in both lines was similar. The cardiomyocytes of LD chickens were larger than those of Ross’s of the same body weight (BW), nevertheless both lines had similar thicknesses of their ventricular walls. The blood capillary density was greater in the LD heart than in that of the Ross heart. The aorta of LD chickens had proportionally; a greater aortic lumen radius, larger numbers of elastic lamellae and more elastic fibers than in Ross chickens. Our results suggest that the heart and aorta of the LD chickens have not been disadvantaged by their intensive genetic selection; furthermore, LD chickens have a better myocardial capillary supply and better aortic mechanical properties than those of Ross chickens.

## Introduction

In response to the increasing demand for safe and economically priced food in large quantities, intensification of the worldwide poultry industry began in the mid-twentieth century. Efficiency was enabled by advances in the breeding, housing and feeding of the birds and an increased knowledge of avian veterinary medicine. Over time, intensive genetic selection resulted in significantly higher egg production by layer type chickens and greater meat yields from broiler chickens. However, with the layer type poultry lines, meat production from male birds is inefficient and uneconomic. Consequently it became common practice, worldwide, to kill one-day-old male hatchlings [1]. For example in Germany, about 46 million one-day-old male chicks from layer genetic lines are killed annually [2].

An alternative to killing one-day-old male layer chickens is to farm dual-purpose chickens, where females lay eggs and males produce meat. Historically traditional dual-purpose breeds are inferior in both egg and meat production to the genetically designed specialized layer and broiler lines [3]. Recently commercial breeding companies have developed new dual-purpose breeds using crosses between meat and layer lines to create breeds such as the Lohmann Dual (LD) that exceed the performance of the late nineteenth and twentieth century dual-purpose breeds such as the Malines and the Schweizerhuhn, particularly in meat production [4].

Genetic selection of domesticated chickens for meat production has produced startling improvements in their growth rates and meat yields. Over the past 60 years growth rates of intensively reared broiler chickens have increased steadily such that a 300% increase in body weight has been engineered from 25 g per day in the 1950s to 100 g per day in the modern bird [5]. Simultaneously, the slaughter age of contemporary broiler chickens has reduced from 9 to 5 weeks and slaughter weight exceeds 2.2 kg [6].

In order to meet the metabolic demands for high muscle mass growth in meat producing birds an optimally functioning cardiovascular system that delivers adequate oxygen to all body cells is critical [7]. The cardiovascular capacity of chickens and of other commercial poultry such as quail and turkeys has been affected greatly by the genetic selection for rapid and high meat production [8]. For example, the hearts of broiler chickens have become progressively smaller in relation to the body mass when compared to their ancestral breeds such as the heritage line from the University of Illinois at Urbana-Champaign (UIUC) [8] and jungle fowl [9]. In addition, modern meat breeds have significantly greater susceptibility to sudden death syndrome and ascites diseases, both strongly linked to morphological changes of the heart and aorta [10]. For instance, the quantity and architecture of the elastic and collagen fibers within the aortic wall directly determines its elasticity and strength. Any significant alteration to these fibers can lead to mechanical and functional changes associated with aortic disease [11].

This study compared the anatomy of the heart and the aorta throughout postnatal growth of a highly selected fast-growing conventional broiler line (Ross 308) to that of the recently developed genetic line of a dual-purpose chicken (Lohmann Dual, LD). For this the thickness of ventricular myocardial walls, cardiomyocyte size, density of myocardial capillaries, aortic wall thickness, aortic diameter, number of aortic elastic lamellae and elastic fiber percentage in the aortic wall were measured.

## Material and methods

### Animals and husbandry

Eighty birds of a broiler line (Ross 308) and 80 birds of a dual-purpose line (Lohmann Dual, LD), were obtained from Lohmann Tierzucht, Cuxhaven, Germany and were kept separately under the same husbandry conditions as described previously [12]. Briefly, chickens had *ad libitum* access to a mash diet and water for the duration of the study. A starter diet (231.5 g protein and 12.6 MJ ME/kg) was fed from hatching to day 14 and then they were fed a grower diet (214.4 g protein and 13 ME/kg) from day 15 to the end of the study.

The study ended when chickens of both lines had reached a live body weight of 2000g, i.e. day 35 for Ross chickens and 63 for LD chickens. The study was authorized by the Animal Welfare Committee: Landesamt für Gesundheit und Soziales, Berlin, Germany, ID: 0236/15.

### Sample collection

On each sample day, for Ross on days 1, 7, 14, 19, 21, 25, 28, 32 and 35, and for LD on days 1, 7, 14, 21, 28, 32, 35, 42, 49, 56 and 63, six birds from each line were selected at random and their live body weight (BW) was measured to an accuracy of 0.1 g using a mechanical scale (Sartorius, Göttingen, Germany). Then the birds were killed by decapitation according to Germany’s animal welfare standards.

### Morphometric analysis of heart

**Fig 1:**
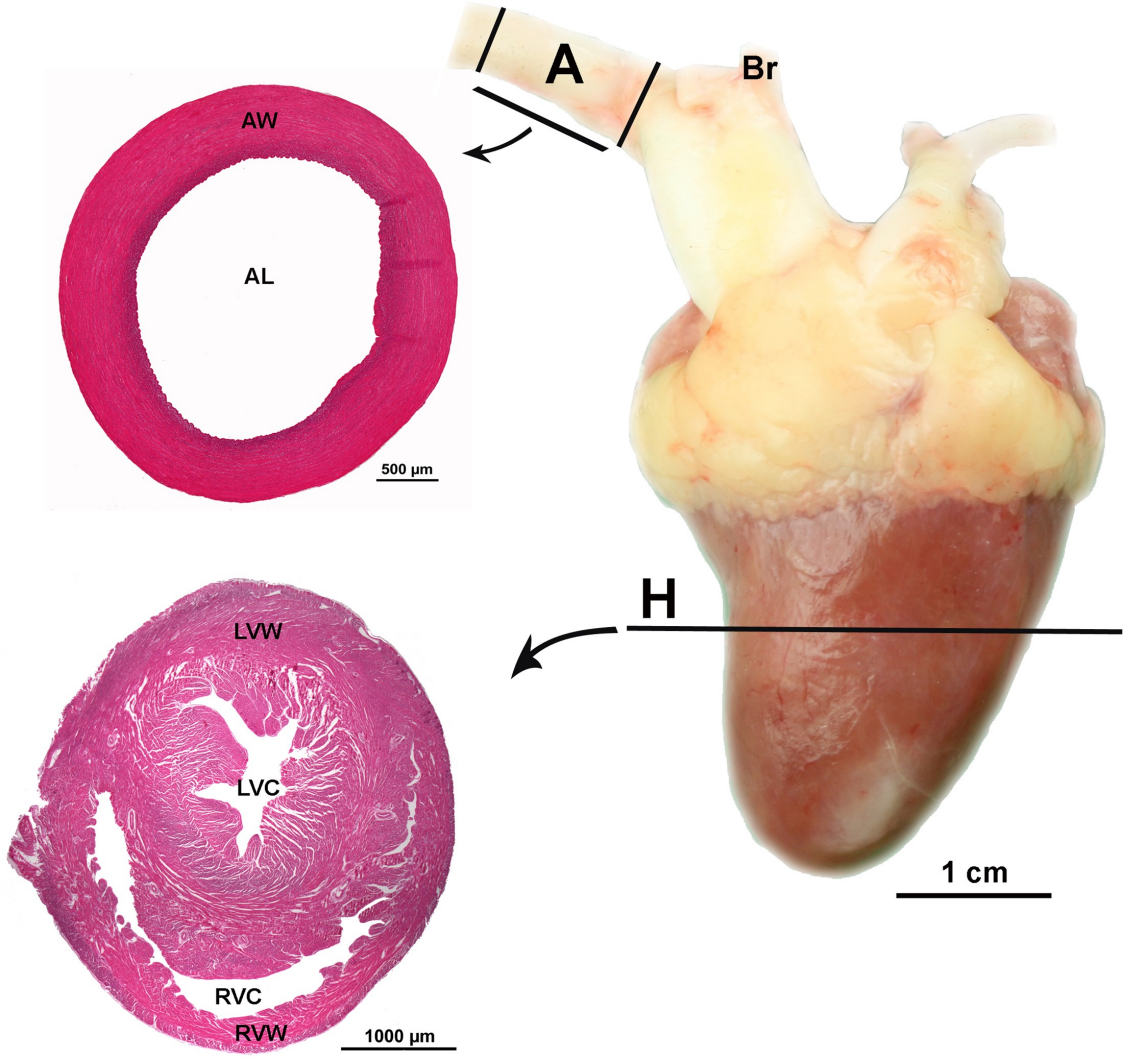
Location of sample collection. Position of the cross section samples from the heart (H) and from the aorta (A); (AL) aortic lumen, (AW) aortic wall, (Br) brachiocephalic arteries, (LVC) left ventricular cavity, (LVW) left ventricular wall, (RVC) right ventricular cavity and (RVW) right ventricular wall of a 63-day-old Lohmann Dual chicken.

Immediately after death, the heart was dissected free from the carcass, weighed to an accuracy of 0.01 g on an electronic laboratory balance (Sauter-Cumulus, Freiburg, Germany) and the relative heart mass (g/100 g BW) determined. A one-centimeter-thick cross section sample was dissected from the midpoint between base and apex of the heart and prepared for morphometric examination (Fig 1). Specimens were washed in 0.9% sodium chloride solution, fixed in phosphate buffered formalin (4%, pH 7, 24 h, room temperature), and then dehydrated in a graded series of ethyl alcohol and embedded in paraffin wax. Eight 5 μm thick transverse serial sections were cut for each bird. Four were stained with Mayer’s hematoxylin and eosin (H&E) for general morphological examination. The other four transverse sections were labelled with the endothelial marker Arachis hypogaea lectin (Peanut agglutinin, PNA) that binds to galactosyl (ß-1,3) N-acetylgalactosamine [13] to examine the capillary network. Two-dimensional morphometric analysis was carried out using an optical microscope (Axioskop, Carl Zeiss, Jena, Germany) and an image analysis system, NIS-Elements AR (Nikon Instruments Inc., U.S.A.).

**Fig 2:**
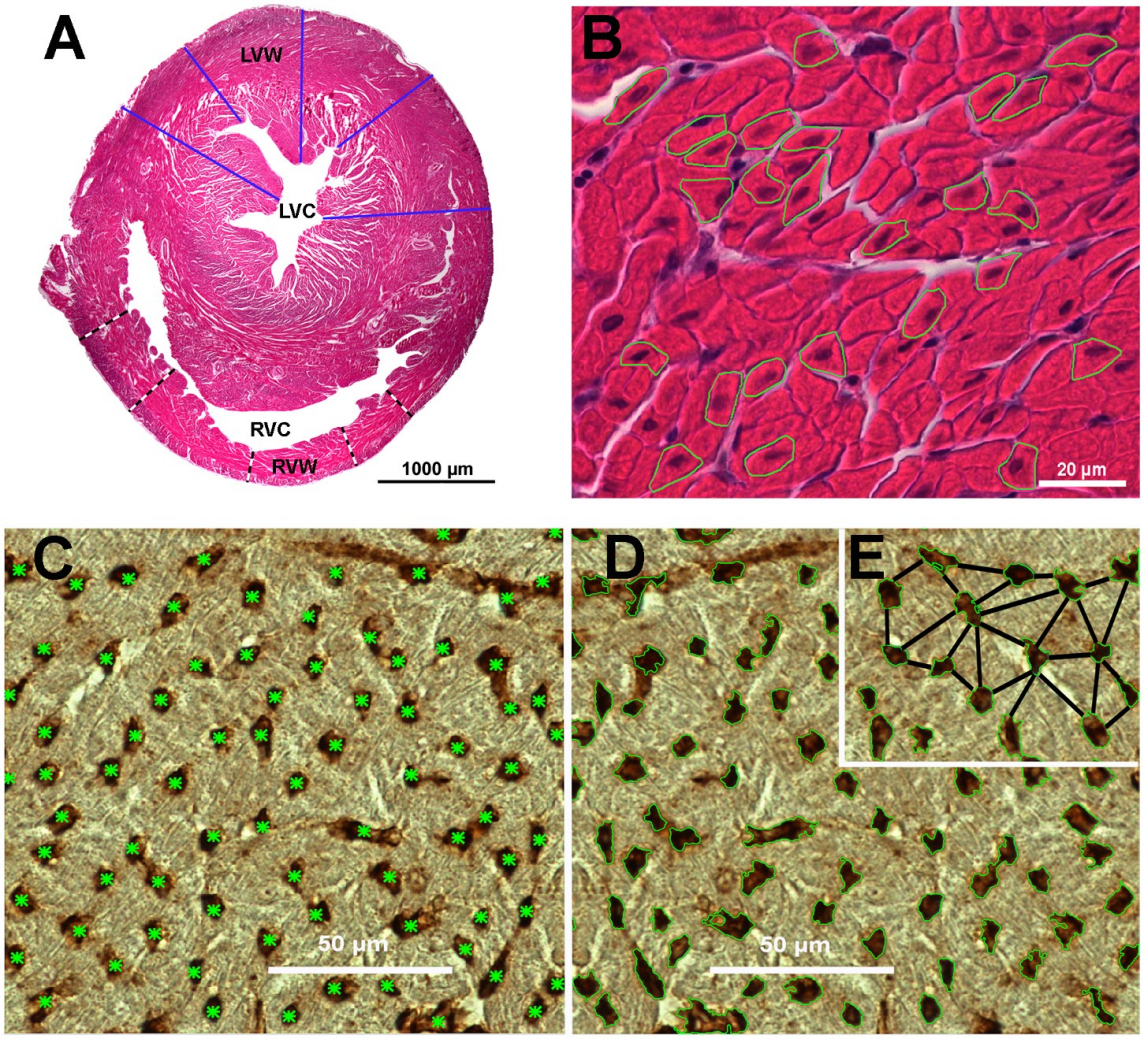
Transverse section of heart ventricular walls of a 63-day-old Lohmann Dual chicken. (A) ventricular wall thicknesses (black-white lines are of the right ventricular wall; blue lines are of the left ventricular wall; (LVC) left ventricular cavity; (LVW) left ventricular wall; (RVC) right ventricular cavity and (RVW) right ventricular wall), (B) representative cardiomyocytes outlined in green, (C) blood capillaries are each indicated by a green asterisk, (D) the area occupied by capillaries is each outlined in green, (E) intercapillary distances; the capillary distances between the nearest neighboring capillaries. A and B stained with H&E. C, D and E stained with Arachis hypogaea Lectin.

Of the four sections stained with H&E the best section that had no artifacts, such as regional tissue shrinkage, wrinkles or cracks was selected and the following measurements were taken:

- The thickness of the left ventricular wall (LVW) was measured at five different locations at a magnification of 12.5× (Fig 2A). Subsequently, the average value of the 5 measurements was calculated. The right ventricular wall (RVW) was measured in the same way (Fig 2A).
- The relative LVW and RVW thicknesses (mm/100g BW) were expressed relative to the bird’s body weight (BW).
- The ratio of the thicknesses of the ventricular walls (right:left).
- Cardiomyocyte size was determined by measuring the cross-sectional area and the diameter of 25 cardiomyocytes in the left ventricular wall, and then the average calculated. The measurement was determined at the level of the nucleus at a magnification of 400× (Fig 2B). Here the outline of each cell was circumscribed manually using the NIS-Elements AR software then the system calculated automatically the diameter (μm) and the cross-sectional area (μm^2^).

Of the four sections labeled with the lectin PNA the best section without artifacts was selected and 5 visual fields (each field = 33000 μm^2^) examined. The following parameters were measured at a magnification of 400×:

- Capillary density:
  1. The number of blood capillary profiles per mm^2^ of field of view. Each capillary profile in the field of view was identified and marked manually by a green asterisk using the imaging software, then the system automatically calculated the total number of the capillary profiles (Fig 2C).
  2. The percentage of the area occupied by capillaries per field of view (Fig 2D).
- The number of the cardiomyocytes per capillary. The cardiomyocytes (only those with a complete nucleus) and the blood capillaries were counted in each visual field, then the number of cardiomyocytes was divided by the number of blood capillary profiles.
- Intercapillary distance (μm) was determined by measuring 25 intercapillary distances in each sample, starting with the capillary located nearest to the center of the field of view. The shortest distance from the outer circumference of this capillary wall was drawn manually to the neighbouring capillaries and from these radially outwards until 25 distances were measured, and the average value was determined according to Al Masri et al, (2017) [14] (Fig 2E).

### Morphometric analysis of aorta

For each bird, a one centimeter wide aortic ring sample was dissected from the aorta immediately distal to the brachiocephalic arteries origin (Fig 1). The specimens were washed in 0.9% sodium chloride solution, fixed in phosphate buffered formalin (4%, pH 7, 24 h, room temperature), dehydrated in a graded series of ethyl alcohol and then embedded in paraffin wax. Eight serial 5 μm thick transverse sections of each sample were cut and four sections stained with H&E and four sections with Weigert’s Resorcin Fuchsin [13]. Two-dimensional morphometric analysis was carried out using an optical microscope (Axioskop, Carl Zeiss, Jena, Germany) and an image analysis system, NIS-Elements AR (Nikon Instruments Inc., U.S.A.).

**Fig 3:**
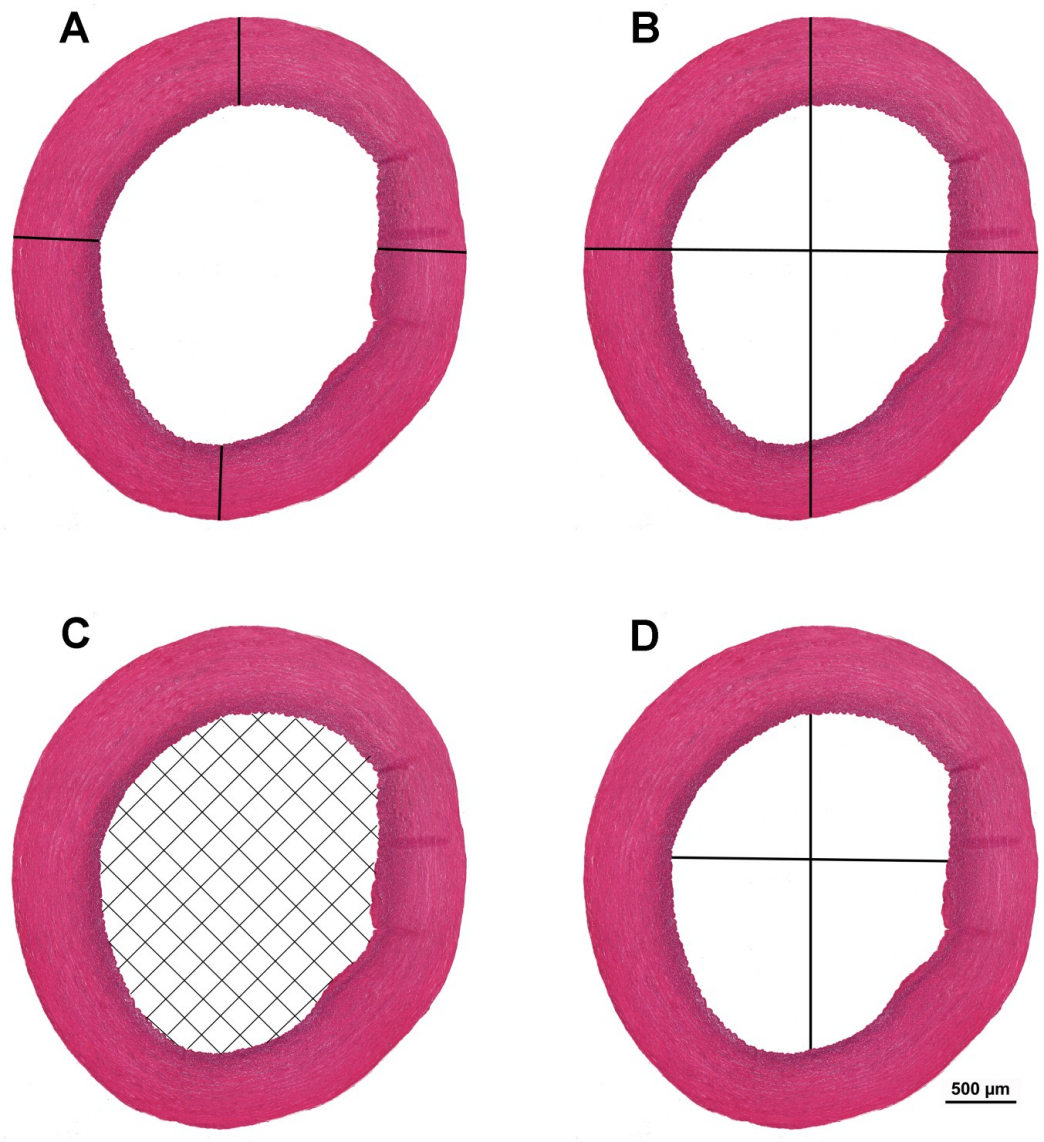
Cross-section of aortic wall of a 7-day-old Lohmann Dual chicken. **(**A) aortic wall thickness, **(**B) aortic diameter, (C) cross sectional area of aortic lumen, (D) diameter of aortic lumen. H&E.

Of the four sections stained with H&E the best section that was free of artifacts was selected and the following parameters were determined for each bird at a magnification of 12.5×:

- Aortic wall thickness (mm) was measured in each sample at four different locations and the mean of the values was calculated (Fig 3A).
- Aortic diameter (mm) was calculated as the mean of the largest and smallest external aortic diameter values (Fig 3B).
- Cross sectional area of the aortic lumen (mm^2^). The aorta inner surface was selected using a colour tool. Based on this selection, the program calculated the surface area of the aortic lumen automatically (Fig 3C).
- Aortic lumen diameter (mm) (Fig 3D).
- Aortic lumen diameter/aortic wall thickness ratio was determined.

**Fig 4:**
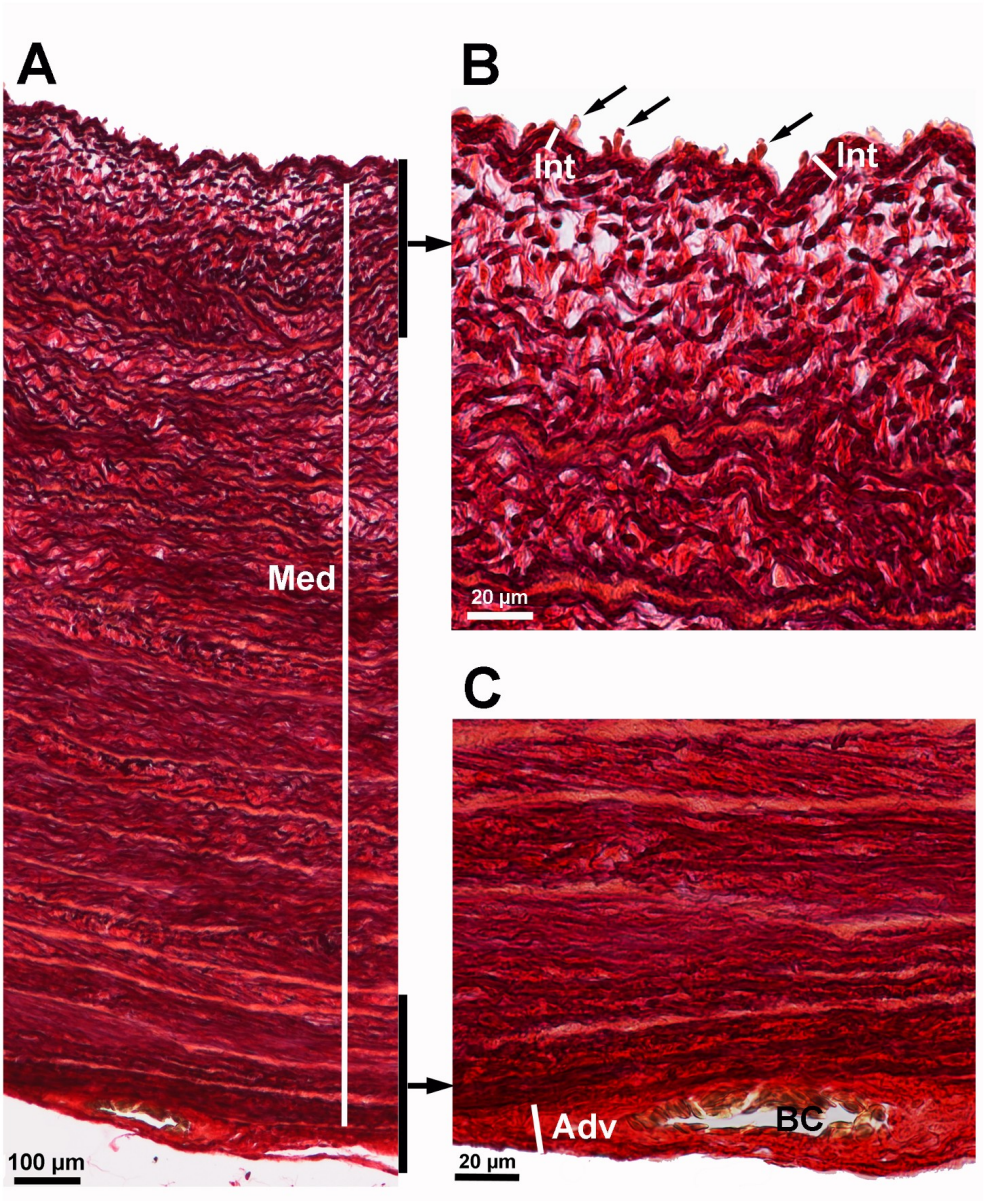
Aortic wall layers of a 35-day-old Lohmann Dual chicken. **(**A) entire aortic wall with tunica media (Med), (B) luminal aspect of the aortic wall showing the tunica intima (Int) and endothelial cells (arrows), (C) outer aspect of the aortic wall showing the tunica adventitia (Adv) and blood capillary (BC). Weigert’s Resorcin Fuchsin.

**Fig 5:**
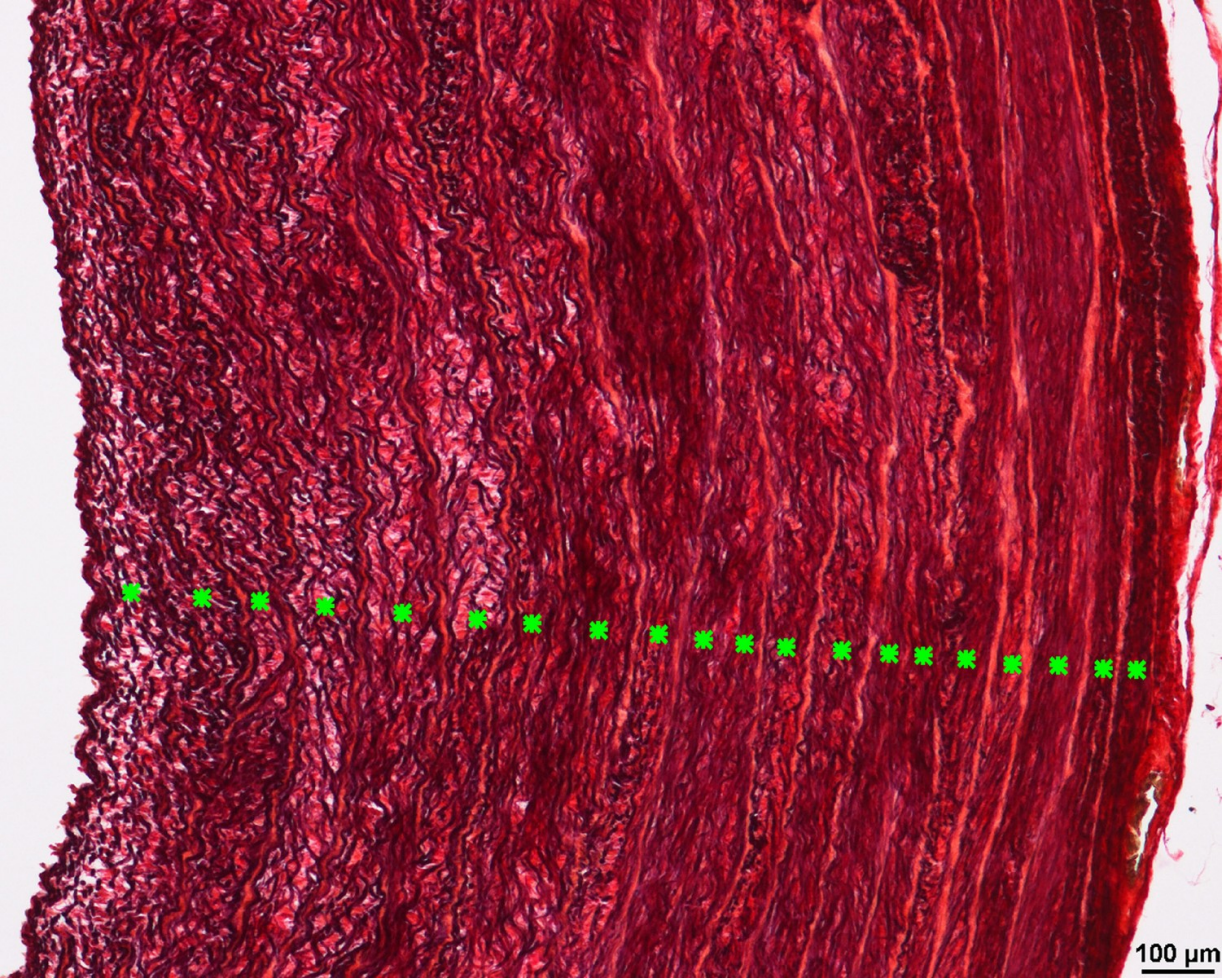
Internal elastic lamellae in the aortic wall of a 35-day-old Lohmann Dual chicken. Each individual elastic lamella is indicated by a green asterisk. Weigert’s Resorcin Fuchsin.

Of the four sections stained with Weigert’s Resorcin Fuchsin the best section without artifacts was selected and the following parameters were determined:

- The thicknesses of the tunica intima (400×), media (50×) and adventitia (400×) of the aorta were measured at four different locations of the histological section. The mean of the measured values was calculated (Fig 4).
- The ratio of the thicknesses of the aortic layers (intima:media:adventitia) was calculated.
- The number of elastic lamellae of the tunica media was counted manually at four different locations of the tunica media under 100× magnification for each bird, then the mean of the measured values was calculated (Fig 5).
- The relative number of elastic lamellae was scaled to a 1 mm aortic wall thickness to allow a comparison across genetic lines.
- The aortic area occupied by elastic fibers (%) was measured by the image analysis system, NIS-Elements AR in 4 randomly chosen visual fields (each field = 0.138 mm^2^, 200 × magnification). The mean of the 4 values was calculated.

### Statistical analysis

Statistical analyses were performed using the statistical package program IBM SPSS Statistics 23 (IBM Corporation, New York, USA). The graphs were generated using the statistical package program JMP Pro 13 (SAS Institute Inc., Cary, USA). Continuous variables are presented as mean ± standard error of the mean (SEM). Comparisons of the two lines of the same age groups were performed using the Mann–Whitney U test. One-way analysis of variance (ANOVA) with a post hoc Least Significant Difference test (LSD) were performed to evaluate the effect of age on the relative mass of the heart and the relative thickness of ventricular walls. To explore the effect of chicken line and BW on heart and aorta measurements, all data collected were regressed against the chicken line and the BW using the log-log regression model. Due to the relationship between some of the data and body weight being non-linear, the body weight and the data were log10-transformed prior to analysis. All statistical analyses were two-sided with significance defined as a p-value of < 0.05.

## Results

### Live body weight

While the broiler line (Ross 308) grew to an average weight of 2.13 kg within 5 weeks post hatching, the dual-purpose chicken line LD took 9 weeks to reach the same average weight. From d 1 to d 35 post hatching, the Ross chickens grew at a rate of 57.7 g/d, and the LD chickens grew at a rate of 22.04 g/d. From d 1 to d 63 post hatching LD grew at a rate of 31.8 g/d. The BW of the Ross chickens significantly exceeded that of the LD chickens at all sampling intervals from d 1 until d 35 (U-test, p < 0.05) (Table 1).

**Table 1:** Live body weight, heart mass and relative heart mass of LD and Ross chicken lines versus day post hatching.

Age (days)	Line (n)	BW (g)	Heart mass (g)	Heart mass (g) per 100g BW
		Mean ± SEM	Mean ± SEM	Mean ± SEM
**1**	**Ross (6)**	52.26 ± 0.94	0.53 ± 0.03	1 ± 0.05
	**LD (6)**	42.45 ± 1.25	0.42 ± 0.02	0.98 ± 0.03
**7**	**Ross (6)**	169.47 ± 7.92	1.65 ± 0.08	0.97 ± 0.03
	**LD (6)**	101.2 ± 3.32	1 ± 0.04	0.99 ± 0.06
**14**	**Ross (6)**	435.35 ± 11.44	3.7 ± 0.19	0.85 ± 0.03
	**LD (6)**	224.77 ± 5.31	1.85 ± 0.04	0.82 ± 0.02
**19**	**Ross (6)**	640.73 ± 45.22	4.62 ± 0.36	0.72 ± 0.02
**21**	**Ross (6)**	746.58 ± 23.81	6.2 ± 0.44	0.83 ± 0.05
	**LD (6)**	329.17 ± 18.8	2.89 ± 0.14	0.89 ± 0.04
**25**	**Ross (6)**	1191.67 ± 37.78	8.5 ± 052	0.71 ± 0.04
**28**	**Ross (6)**	1221 ± 44.5	8.94 ± 0.6	0.73 ± 0.03
	**LD (6)**	575.33 ± 25.54	4.25 ± 0.29	0.74 ± 0.02
**32**	**Ross (6)**	1677.83 ± 70.52	11.01 ± 0.7	0.65 ± 0.02
	**LD (6)**	754.17 ± 38.3	5.49 ± 0.27	0.74 ± 0.04
**35**	**Ross (6)**	2013.17 ± 58.26	12.58 ± 0.26	0.63 ± 0.01
	**LD (6)**	791.67 ± 23.85	6.03 ± 0.21	0.76 ± 0.02
**42**	**LD (6)**	1130.5 ± 24.15	7.7 ± 0.18	0.68 ± 0.02
**49**	**LD (6)**	1522.5 ± 46.02	10.49 ± 0.58	0.69 ± 0.03
**56**	**LD (6)**	1817.33 ± 54.79	11.77 ± 0.53	0.65 ± 0.03
**63**	**LD (6)**	2011.83 ± 74.66	12.27 ± 0.59	0.61 ± 0.02

BW: live body weight; LD: Lohmann Dual; Line: genetic line; n: animal number; Ross: Ross 308; SEM: standard error of the mean.

### Macroscopic examination of the heart

#### Heart mass and relative heart mass

**Fig 6:**
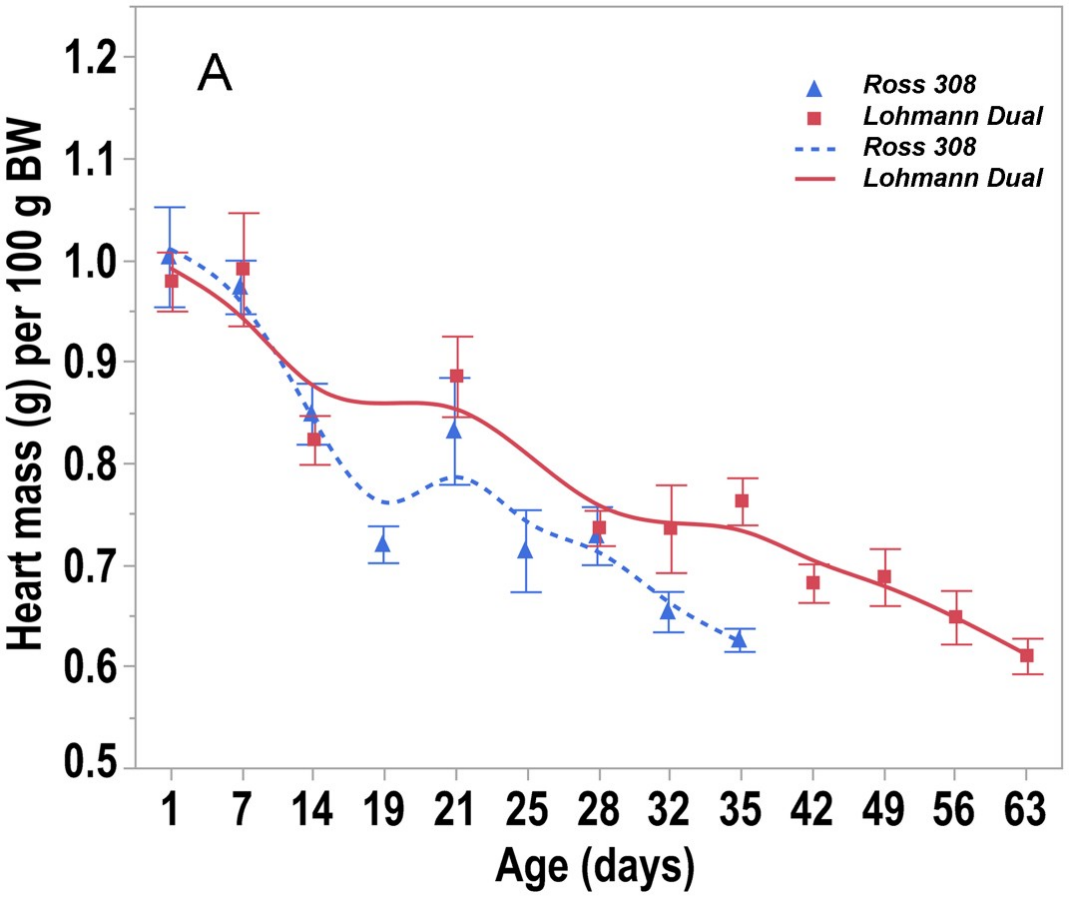
Trendlines of the changes in relative heart mass versus day post hatching. Bars refer to mean ± standard error of the mean of the chicken samples at each time interval.

The heart mass increased steadily with age in both lines. From d 1 to d 35 post hatching, the Ross birds’ heart grew at a rate of 0.344 g/d, whereas the LD birds’ heart grew by 0.16 g/d over the same period, and by a rate of 0.188 g/d from d 1 to d 63. The mass of the heart in LD chickens was significantly lower than that of Ross chickens in all comparable age groups between d 1 and d 35 post hatching (U-test, p < 0.05) (Table 1). When heart mass was expressed relative to body weight, LD chickens had a higher relative heart mass than Ross chickens only at d 35 (U-test, p = 0.004). The relative heart mass in both lines decreased with increasing age. The relative heart mass of LD chickens decreased significantly between d 7 and d 14 as well as between d 21 and d 28 by 20% (LSD, p ≤ 0.001). In Ross chickens, the decrease in the relative heart mass was between d 7 and d 14 by 15% (LSD, p = 0.011) and by 18% between d 14 and d 19 (LSD, p = 0.009) (Fig 6, Table 1). Regression analysis showed that when the birds were of the same BW, the chicken line had no effect on the heart mass (p = 0.091), whereas the body weight had an effect on the heart mass (p < 0.001), adjusted R^2^ = 0.99.

#### Ventricular myocardial walls

In both chicken lines, the absolute ventricle wall thicknesses increased with each time interval, however the relative thickness of the ventricle walls decreased with age. The thickness of the LVW increased similarly in both lines up to d 14 without any differences between LD and Ross chickens. From d 21 onwards, the LVW was found to be significantly thinner in LD chickens compared to that of Ross chickens (U-test, p < 0.05). However, except at hatching, Lohmann Dual chickens had a thicker relative LVW than Ross chickens did in all age groups (U-test, p < 0.05).

The RVW was thinner in LD than in Ross chickens only at d 14 and d 32 (U-test, p < 0.05). At all other times the thickness did not differ between the two chicken lines. However, the relative thickness of RVW was greater in LD chickens than in Ross’s in all age matched groups (U-test, p < 0.05), except at d 14 and d 28 (Table 2). Regression analysis showed that when the birds had an equal BW, the chicken line had no effect on ventricular wall thicknesses (LVW and RVW). However, body weight had an effect (p < 0.001), adjusted R^2^ = 0.89 and 0.40 for LVW and RVW, respectively. The ratio of the thickness of the ventricular walls (right:left) at d 1 and d 35 was significantly lower in LD chickens (1:2.5, 1:2.9) than in Ross chickens (1:3.4, 1:4.1). The overall ratio was 1:4.1 for Ross and 1:3.7 for LD (Table 2).

**Table 2:** Ventricular wall measurements over time of both chicken lines.

Age (days)	Line (n)	RVW thickness (mm)	LVW thickness (mm)	RVW thickness (mm) per 100g BW	LVW thickness (mm) per 100g BW	LVW:RVW ratio
		Mean ± SEM	Mean ± SEM	Mean ± SEM	Mean ± SEM	Mean ± SEM
**1**	**Ross (6)**	0.46 ± 0.06	1.44 ± 0.08	0.87 ± 0.12	2.77 ± 0.19	3.4 ± 0.34
	**LD (6)**	0.51 ± 0.02	1.27 ± 0.06	1.32 ± 0.1	3 ± 0.06	2.5 ± 0.17
**7**	**Ross (6)**	0.4 ± 0.02	1.79 ± 0.12	0.24 ±0.02	1.06 ± 0.08	4.51 ± 0.24
	**LD (6)**	0.45 ± 0.03	1.62 ± 0.15	0.45 ± 0.02	1.6 ± 0.13	3.65 ± 0.41
**14**	**Ross (6)**	0.94 ± 0.11	2.08 ± 0.35	0.21 ± 0.02	0.47 ± 0.08	2.37 ± 0.48
	**LD (6)**	0.62 ± 0.05	2.01 ± 0.05	0.28 ± 0.02	0.89 ± 0.03	3.32 ± 0.2
**19**	**Ross (6)**	0.67 ± 0.06	2.55 ± 0.24	0.11 ± 0.01	0.41 ± 0.04	4.1 ± 0.64
**21**	**Ross (6)**	0.68 ± 0.07	3.02 ± 0.17	0.09 ± 0.01	0.4 ± 0.02	4.64 ± 0.48
	**LD (6)**	0.54 ± 0.05	2.26 ± 0.09	0.16 ± 0.01	0.7 ± 0.04	4.28 ± 0.3
**25**	**Ross (6)**	0.86 ± 0.17	3.5 ± 0.08	0.07 ± 0.02	0.3 ± 0.01	4.67 ± 0.65
**28**	**Ross (6)**	0.89 ± 0.15	3.59 ± 0.24	0.07 ± 0.01	0.29 ± 0.01	4.45 ± 0.54
	**LD (6)**	0.74 ± 0.12	2.7 ± 0.05	0.13 ± 0.03	0.47 ± 0.02	4.13 ± 0.6
**32**	**Ross (6)**	0.79 ± 0.05	3.44 ± 0.1	0.05 ± 0	0.2 ±0.01	4.44 ± 0.31
	**LD (6)**	0.55 ± 0.03	2.27 ± 0.08	0.07 ± 0	0.3 ± 0.02	4.17 ± 0.3
**35**	**Ross (6)**	1.05 ± 0.06	4.28 ± 0.06	0.05 ± 0	0.21 ± 0.01	4.14 ± 0.25
	**LD (6)**	1.07 ± 0.1	2.92 ± 0.15	0.14 ± 0.01	0.37 ± 0.03	2.9 ± 0.4
**42**	**LD (6)**	0.94 ± 0.07	2.78 ± 0.19	0.08 ± 0.01	0.25 ± 0.02	3.06 ± 0.35
**49**	**LD (6)**	0.82 ± 0.06	3.18 ± 0.1	0.05 ± 0	0.21 ± 0.01	3.95 ± 0.31
**56**	**LD (6)**	0.9 ± 0.11	3.13 ± 0.23	0.05 ± 0	0.17 ± 0.02	3.75 ± 0.65
**63**	**LD (6)**	0.81 ± 0.05	3.68 ± 0.16	0.04 ± 0	0.18 ± 0.01	4.61 ± 0.26

LD: Lohmann Dual; LVW: left ventricular wall; Line: genetic breed; n: animal number; Ross: Ross 308; RVW: right ventricular wall; SEM: standard error of the mean.

### Microscopic examination of the heart

#### Size of the cardiomyocytes

**Fig 7:**
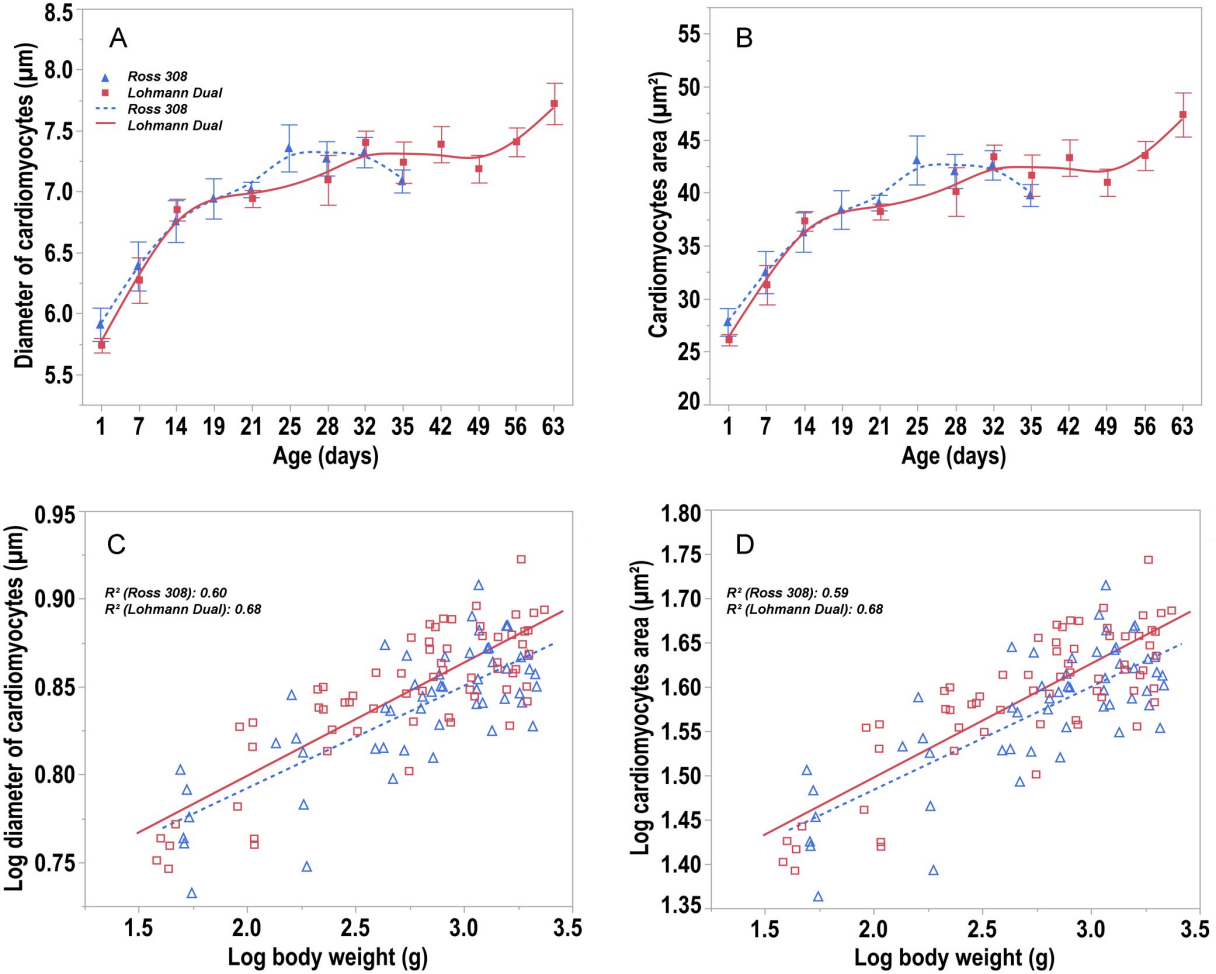
Trendlines of the changes in diameter and cross sectional area of cardiomyocytes versus day post hatching. (A) diameter of cardiomyocytes and (B) cross sectional area of cardiomyocytes versus day post hatching for Ross and LD chicken lines. Bars refer to mean ± standard error of the mean of the chicken samples at each time interval. (C) allometric plot: log transformed diameter of cardiomyocytes and (D) cross sectional area of cardiomyocytes versus log of body weight post hatching for Ross and LD chicken lines. Symbols represent each individual value for each chicken line.

From day 1 to day 35, the cardiomyocytes’ diameter increased by 20% for LD chickens and 26.35% for Ross chickens whilst the cardiomyocytes’ cross-sectional area increased by 43% (LD) and 59.5% (Ross) (Fig 7A and 7B).

No differences between the two chicken lines were found at any age. However, regression analysis showed that the cardiomyocyte diameter was 1.2% greater (p = 0.006) (Fig 7C) and their cross sectional area was about 2.3% larger (p = 0.007) (Fig 7D) in LD chickens compared to those of Ross chickens of the same body weight, R^2^ = 0.65 and 0.65, respectively.

#### Myocardial capillary density

In comparisons of the myocardium in both chicken lines at hatching, the percentage area occupied by capillaries and the number of capillary profiles in the myocardium, were greater in LD chickens than in Ross chickens (U-test, p = 0.009). From d 1 to d 7, the means of these values did not change in LD chickens, whereas they increased in Ross chickens (LSD, p ≤ 0.001) resulting in no difference between LD and Ross chickens at d 7. Subsequently, from d 14 to d 28 the values did not change in each chicken line and there was no difference between LD and Ross chickens. At 32 and 35 days the myocardium of LD chickens had larger areas occupied by capillaries as well as greater number of capillary profiles than did Ross chickens (U-test, p < 0.05) (Table 3). The intercapillary distance in the myocardium of LD chickens was shorter than in Ross chickens only at d 35 (U-test, p = 0.045). At all other ages there were no differences between both lines (Table 3). The number of cardiomyocytes supplied by one capillary decreased over the time of the study in both chicken lines. The number of cardiomyocytes per capillary did not differ between both chicken lines of the same age, except at 32 days. At this age, one blood capillary supplied 2.36 cardiomyocytes in LD chickens and 3.29 in Ross chickens (U-test, p = 0.013). This number decreased from 3.9 to 2.4 in LD chickens and from 3.7 to 3.2 in Ross chickens over the time line of the study (Table 3).

**Table 3:** Ventricular capillary and cardiomyocyte measurements over time of LD and Ross chicken lines.

Age (days)	Line (n)	Number of capillary profiles per mm^2^	Area occupied by capillaries %	Cardiomyocyte per capillary	Intercapillary distance (μm)
		Mean ± SEM	Mean ± SEM	Mean ± SEM	Mean ± SEM
**1**	**Ross (6)**	3123.44 ± 83.85	4 ± 0	3.73 ± 0.23	10.38 ± 0.32
	**LD (6)**	3734.42 ± 178.18	7 ± 0	3.91 ± 0.25	11.18 ± 0.52
**7**	**Ross (6)**	3857.02 ± 161.73	7 ± 0	3.19 ± 0.21	10.76 ± 0.54
	**LD (6)**	3955.02 ± 140.06	7 ± 0	3.61 ± 0.4	10.64 ± 0.76
**14**	**Ross (6)**	3546.02 ± 182.18	7 ± 0.01	2.52 ± 0.21	10.52 ± 0.71
	**LD (6)**	4069.02 ± 194.27	9 ± 0.01	3.04 ± 0.32	10.21 ± 0.34
**19**	**Ross (6)**	3926.42 ± 111.97	9 ± 0.01	3.04 ± 0.24	9.87 ± 0.54
**21**	**Ross (6)**	3884.42 ± 240.99	8 ± 0.01	2.6 ± 0.17	10.93 ± 0.42
	**LD (6)**	3736.82 ± 166.22	9 ± 0.01	2.43 ± 0.13	10.58 ± 0.57
**25**	**Ross (6)**	3968.02 ± 226.02	9 ± 0.01	2.7 ± 0.15	11.17 ± 0.36
**28**	**Ross (6)**	3406.81 ± 122.68	8 ± 0.01	2.68 ± 0.08	10.44 ± 0.44
	**LD (6)**	3722.02 ± 110.93	8 ± 0	2.71 ± 0.17	9.04 ± 0.72
**32**	**Ross (6)**	3231.01 ± 160.69	7 ± 0	3.29 ± 0.19	9.92 ± 0.68
	**LD (6)**	3749.02 ± 111.57	9 ± 0.01	2.36 ± 0.2	9.17 ± 0.37
**35**	**Ross (6)**	3860.02 ± 368.08	6 ± 0	2.46 ± 0.11	10.4 ± 0.47
	**LD (6)**	3563.02 ± 168.18	8 ± 0	2.89 ± 0.21	9.13 ± 0.41
**42**	**LD (6)**	3651.02 ± 150.29	10 ± 0	2.48 ± 0.14	8.76 ± 0.2
**49**	**LD (6)**	3783.02 ± 109.91	9 ± 0.01	2.3 ± 0.1	8.9 ± 0.48
**56**	**LD (6)**	3479.01 ± 112.65	9 ± 0	2.45 ± 0.12	9.13 ± 0.31
**63**	**LD (6)**	3605.02 ± 119.03	10 ± 0.01	2.33 ± 0.1	10.46 ± 0.61

LD: Lohmann Dual; Line: genetic line; n: animal number; Ross: Ross 308; SEM: standard error of the mean.

### Microscopic examination of the aorta

#### Aortic wall thickness

The aortic wall thickness increased with age in both lines. The aortic wall thickness showed no differences between LD chickens and Ross chickens at hatching and at d 7. Thereafter, the aortic wall in LD chickens was significantly thinner than in Ross chickens in all age matched groups (Table 4).

**Table 4:** Aortic wall measurements over time of LD and Ross chicken lines.

Age (days)	Line (n)	Wall thickness of aorta (mm)	Layers of aortic wall			Intima:media:adventitia ratio	
			Int thickness (μm)	Med thickness (μm)	Adv thickness (μm)	Med:Int	Adv:Int
		Mean ± SEM	Mean ± SEM	Mean ± SEM	Mean ± SEM	Mean ± SEM	Mean ± SEM
**1**	**Ross (6)**	0.47 ± 0.01	2.14 ± 0.05	456 ± 12.77	12.98 ± 0.25	212.9 ± 3.25	6.1 ± 0.1
	**LD (6)**	0.43 ± 0.06	2.29 ± 0.05	440.48 ± 61.87	12.99 ± 0.07	191.12 ± 24.8	5.7 ± 0.13
**7**	**Ross (6)**	0.6 ± 0.05	2.48 ± 0.08	623.55 ± 45.65	13.06 ± 0.1	250.1 ± 13.94	5.3 ± 0.18
	**LD (6)**	0.59 ± 0.04	2.42 ± 0.07	571.03 ± 39.86	13.01 ± 0.07	236.6 ± 16.83	5.35 ± 0.15
**14**	**Ross (6)**	0.8 ± 0.02	2.57 ± 0.04	781.28 ± 20.56	13.19 ± 0.1	303.8 ± 5.83	5.14 ± 0.11
	**LD (6)**	0.63 ± 0.02	2.68 ± 0.06	611.55 ± 23.88	13.23 ± 0.05	228.83 ± 7.6	5 ± 0.11
**19**	**Ross (6)**	0.87 ± 0.05	2.75 ± 0.07	852.02 ± 47.65	13.26 ± 0.06	312.2 ± 22.33	4.84 ± 0.12
**21**	**Ross (6)**	0.92 ± 0.04	2.71 ± 0.05	904.69 ± 37.91	13.15 ± 0.07	344.5 ± 10.3	4.83 ± 0.09
	**LD (6)**	0.69 ± 0.03	2.82 ± 0.08	673.86 ± 43.65	13.20 ± 0.11	239.46 ± 16,83	4.7 ± 0.14
**25**	**Ross (6)**	0.95 ± 0.05	2.85 ± 0.05	934.58 ± 55.56	13.39 ± 0.11	322.31 ± 20.81	4.7 ± 0.11
**28**	**Ross (6)**	0.99 ± 0.04	2.81 ± 0.03	970.28 ± 39.92	13.19 ± 0.1	344.56 ± 11.56	4.7 ± 0.07
	**LD (6)**	0.77 ± 0.05	2.71 ± 0.04	752.6 ± 49.48	13.44 ± 0.12	277.52 ± 16.7	5 ± 0.05
**32**	**Ross (6)**	0.94 ± 0.04	2.79 ± 0.06	927.25 ± 37.45	13.42 ± 0.07	331.58 ± 9.7	4.82 ± 0.11
	**LD (6)**	0.73 ± 0.03	2.77 ± 0.02	737.60 ± 28.36	13.33 ± 0.12	266.3 ± 10.4	4.81 ± 0.04
**35**	**Ross (6)**	1.01 ± 0.03	2.88 ± 0.06	969.49 ± 33.6	13.2 ± 0.05	336.6 ± 19	4.6 ± 0.1
	**LD (6)**	0.75 ±0.01	2.89 ± 0.1	737.02 ± 13.57	13.46 ± 0.1	256.18 ± 9.62	4.7 ± 0.16
**42**	**LD (6)**	0.88 ± 0.03	3.12 ± 0.05	867.48 ± 28.81	13.67 ± 0.07	279.1 ± 12.76	4.4 ± 0.1
**49**	**LD (6)**	0.87 ± 0.02	3.17 ± 0.07	855.24 ± 3	13.94 ± 0.08	271.13 ± 14.74	4.4 ± 0.12
**56**	**LD (6)**	0.93 ± 0.04	3.18 ± 0.04	915.73 ± 46.83	13.96 ± 0.12	288.6 ± 14.3	4.4 ± 0.1
**63**	**LD (6)**	0.9 ± 0.03	3.16 ± 0.01	878.22 ± 34.37	13.98 ± 0.07	278.1 ± 10.8	4.43 ± 0.02

Adv: tunica adventitia; Int: tunica intima; LD: Lohmann Dual; Line: genetic line; Med: tunica media; n: animal number; Ross: Ross 308; SEM: standard error of the mean.

#### Layers of aortic wall

Analysis of the individual aortic wall layers indicated no difference in the thickness of the tunica intima and adventitia between the two chicken lines in all age matched groups. However, the thickness of the tunica media from d 14 to d 32 was lower in LD chickens than in Ross chickens (U-test p ≤ 0.018) (Table 4). Furthermore, the intima:media ratio was greater in LD than in Ross birds between d 14 and d 32 (U-test p ≤ 0.018). The overall ratio of the thicknesses of the aortic layers (intima:media:adventitia) was 1:256:4.8 for LD and 1:302:4.9 for Ross (Table 4).

#### Aortic lumen radius

The radius of the aortic lumen increased continuously with age in both chicken lines. LD chickens had a lower aortic lumen radius than Ross chickens on d 28 and d 35 (U-test, p ≤ 0.05) (Table 5). However, when both chicken lines had the same BW, the radius of the aortic lumen was 7.2% greater in LD chickens than in the Ross chickens, p < 0.001, R^2^ = 0.85.

**Table 5:** Radius of aortic lumen, ratio of aortic lumen diameter/aortic wall thickness and cross sectional area of aortic lumen. All versus day post hatching.

Age (days)	Line (n)	Radius of aortic lumen (mm)	Aortic lumen diameter/aortic wall thickness ratio	Cross sectional area of aortic lumen (mm^2^)
		Mean ± SEM	Mean ± SEM	Mean ± SEM
**1**	**Ross (6)**	0.25 ± 0.03	1.09 ± 0.14	0.2 ± 0.04
	**LD (6)**	0.35 ± 0.04	1.72 ± 0.21	0.4 ± 0.11
**7**	**Ross (6)**	0.48 ± 0.05	1.63 ± 0.22	0.78 ± 0.14
	**LD (6)**	0.48 ± 0.07	1.65 ± 0.22	0.87 ± 0.25
**14**	**Ross (6)**	0.85 ± 0.0.3	2.15 ± 0.12	2.25 ± 0.21
	**LD (6)**	0.80 ± 0.03	2.6 ± 0.18	2.12 ± 0.16
**19**	**Ross (6)**	1.06 ± 0.07	2.49 ± 0.22	3.6 ± 0.5
**21**	**Ross (6)**	1.06 ± 0.07	2.3 ± 0.1	3.7 ± 0.5
	**LD (6)**	1 ± 0.05	2.98 ± 0.25	3.16 ± 0.3
**25**	**Ross (6)**	1.35 ± 0.08	2.86 ± 0.15	5.9 ± 0.7
**28**	**Ross (6)**	1.45 ± 0.02	2.96 ± 0.15	6.8 ± 0.24
	**LD (6)**	1.23 ± 0.4	3.27 ± 0.19	4.74 ± 0.33
**32**	**Ross (6)**	1.36 ± 0.08	2.94 ± 0.27	6.1 ± 0.7
	**LD (6)**	1.32 ± 0.09	3.16 ± 0.19	5.53 ± 0.9
**35**	**Ross (6)**	1.62 ± 0.06	3.25 ± 0.2	8.3 ± 0.5
	**LD (6)**	1.36 ± 0.07	3.62 ± 0.21	6.21 ± 0.7
**42**	**LD (6)**	1.53 ± 0.04	3.48 ± 0.2	7.25 ± 0.34
**49**	**LD (6)**	1.38 ± 0.04	3.2 ± 0.05	5.82 ± 0.32
**56**	**LD (6)**	1.5 ± 0.05	3.23 ± 0.11	7.1 ± 0.4
**63**	**LD (6)**	1.52 ± 0.06	3.41 ± 0.14	7.36 ± 0.76

LD: Lohmann Dual; Line: genetic line; n: animal number; Ross: Ross 308; SEM: standard error of the mean.

#### Aortic lumen diameter/aortic wall thickness ratio

The ratio of aortic lumen diameter/aortic wall thickness increased continuously with age in both chicken lines and was greater in LD chickens than in Ross chickens at d 21 (U-test, p = 0.025). There were no significant differences between the chicken lines in all other age groups (Table 5).

#### Aortic lumen area

The cross sectional area of the aortic lumen increased with age in both chicken lines. There was no difference between both chicken lines in the age matched groups, except on d 28, when the aorta of LD chickens had a smaller lumen than that of Ross chickens (U-test, p = 0.011). According to the regression analysis, the chicken line as well as the body weight had an effect on the aortic lumen. On average, the aorta of LD chickens had a 14% greater luminal cross sectional area than that of Ross chickens of the same body weight, p < 0.001, R^2^ = 0.84. (Table 5).

#### Elasticity of aorta

**Fig 8:**
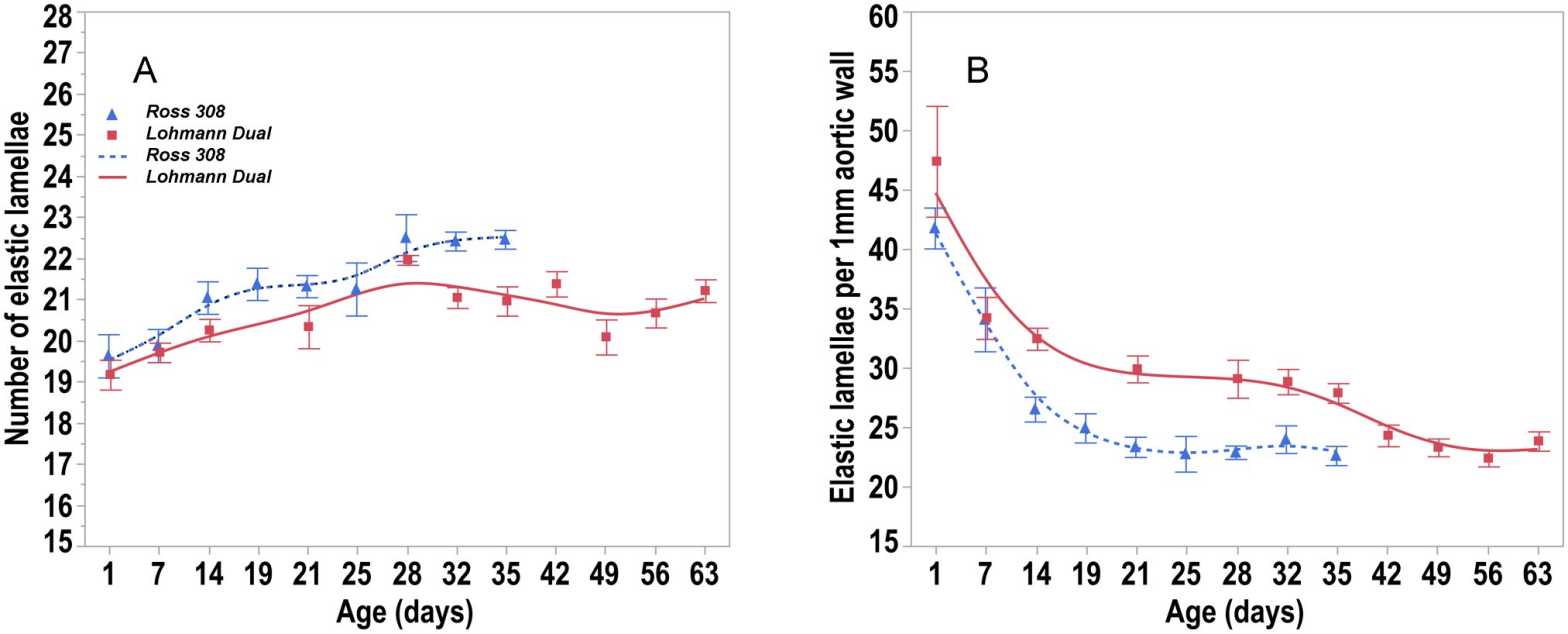
Trendlines of the changes in number of elastic lamellae versus day post hatching. (A) number of elastic lamellae and (B) number of elastic lamellae per 1mm aortic wall versus days post hatching for Ross and LD chicken lines. Bars refer to mean ± standard error of the mean of the chicken samples at each time interval.

The area occupied by elastic fibers in the tunica media decreased steadily with age in both chicken lines. It was greater in the LD chickens’ aorta than in the Ross chickens’ aorta by 13.3% at d 21 and by 50% at d 35 (U-test, p < 0.05). The number of elastic lamellae did not change with age in both lines. The number of elastic lamellae in the aortic tunica media ranged from 19.2 to 22.2 in LD chickens and from 19.6 to 22.5 in Ross chickens. There were no significant differences between LD chickens and Ross chickens (Fig 8A). Nevertheless, the number of elastic lamellae per 1 mm of the aortic wall thickness was higher in LD chickens than in Ross chickens from d 14 onward (U-test, p < 0.05) (Fig 8B).

## Discussion

Our findings confirm other studies that, at all ages, the body weight of Ross chickens was significantly higher than that of LD chickens. In the present investigation the BW at slaughter reached by LD chickens was lower than that of broiler lines such as Ross 308 and Ross MP3 [4,15]. However, at 35 days of age the BW attained by LD males in our study was twice the body weight (791 g) of similar aged Leghorn layer chickens (394 g) reported in a comparable study [16]. At 63 days, the slaughtered LD males in the present experiment had a BW of 2012 g, making them heavier than the Lohmann Brown layer males reported by Habig et al, (2016), that at the same age weighed just 1233 g [15]. The slaughter weight of contemporary broiler chickens at 5 weeks of age exceeds 2.2 kg [6], whereas in commercial layer hens killed when 73 weeks old, the average body weight of spent hens is 1.9 kg [17]. Due to the large differences in the body growth traits of intensively genetically selected broiler breeds to that of layer breeds, it does not appear possible to breed dual-purpose male chickens to have comparable meat production performance levels to those of the specialized lines.

Our observations highlight that, there were no differences in the growth patterns of the heart of both lines. Although the absolute heart mass of LD chickens was lower than that of Ross chickens over the entire observation period, the relative heart mass in both lines was similar and decreased continuously with age. Our results are in agreement with the findings of similar comparative studies [14,18,19] that reported that the relative heart mass of poultry diminishes with age. However, in these studies the relative heart mass of unselected chicken lines was greater than that of modern meat-type chicken lines. Schmidt et al. (2009) in a comparative study of heart growth in a modern broiler line (Ross 708) to that of the UIUC heritage chickens, where no selection had occurred for over 65 years, found no difference in the relative heart mass between these two lines until 14 days post hatching [8]. They found that after day 14 the relative heart mass of the broiler line became significantly less than that of the heritage line. This drop in the relative heart mass in the broiler lines is, most probably, due to their much larger somatic muscle mass. Presumably, the reduced relative heart mass in the broiler lines could result in diminished cardiac capacity compromising their rapid somatic muscle growth and ultimately the welfare of individual birds [20,21].

Regression analysis of our data showed no difference between the two lines in terms of the allometric growth of the ventricular walls. Nevertheless, the relative thickness of left and right ventricular walls was greater in LD than in Ross chickens. This could be due to the positive correlation between ventricular wall thicknesses and BW growth in LD chickens, whereas in Ross chickens the development of the ventricular wall thickness lags behind BW growth. Many studies report similar findings in slow-growing; chicken [16] and turkey breeds [14] which have relatively thicker ventricular walls than those of fast-growing breeds.

Cardiomyocytes are the individual functional units of cardiac muscle, providing the contractile power of the heart. Domestic chickens, ducks and pheasants all have cardiomyocyte diameters ranging from 3.5 to 6.3 μm [22–24]. In our study the diameter and the cross-sectional area of cardiomyocytes were 7.7 μm and 47.4 μm^2^, respectively, in LD birds compared with 7.4 μm and 39.8 μm^2^, respectively in Ross birds. However, Li et al. (1997) who isolated the cardiomyocytes enzymatically from layer chickens’ hearts recorded the cardiomyocyte diameter to be 8.7 μm and a cross-sectional area of 62.3 μm^2^.

When LD and Ross chickens reach the same BW, the cardiomyocytes of LD chickens were about 1.2% larger in diameter and about 2.3% larger in the cross sectional area than those of Ross chickens. Nevertheless both lines had similar ventricular wall thicknesses.

It has been shown, that at the time of hatching, cardiac myocytes cease dividing, but do increase in size as the birds age [25]. Anatskaya et al. (2002) demonstrated in 31 different avian species that the increase in the ventricular wall thickness as the birds age was due to an increase of the size of the cardiomyocytes [26]. In the present study, where the ventricular walls of both genetic lines remained similar in age matched pairs, the number of cardiomyocytes were similar, but those of the LD chickens were larger. This suggests that LD chickens have lower volumes of ventricular wall connective tissue than the Ross chickens.

The number and size of the capillary profiles supplying the cardiomyocytes as well as the diffusion distances between capillaries are important descriptive measures of the oxygen and nutrient supply of the myocardium. In the present study although the LD chickens’ heart had less mass than the broilers’ heart in all age groups, more of the heart area measured was occupied by capillaries from day 28 onward, at which time the area occupied by capillaries in Ross heart decreased rapidly. Similar to our findings, wild-type turkeys had a higher capillary density compared to highly selected meat-type turkeys [14]. Our observations confirm that capillary density is negatively correlated with total heart weight, i.e. larger hearts of fast growing genetically selected modern broilers have a lower capillary density which could be a contributor to hypoxia [27].

Michel et al. (1972) reported a relationship of one capillary to four cardiomyocytes in domestic chickens [22]. In our study although the number and the intercapillary distance of blood capillary profiles did not change significantly over time, the number of cardiomyocytes supplied by one capillary decreased from 3.9 to 2.4 in LD chickens and from 3.7 to 3.2 in Ross chickens over the time line of the study. This is probably due to the increase in cardiomyocyte size over time, whilst the number of capillary profiles per unit area remain constant [27].

Generally, birds have a higher blood pressures than mammals of comparable body weights due to the relatively larger volumes of their heart chambers and greater stroke volumes resulting in larger cardiac outputs than found in mammals [28]. Furthermore, the increase in blood pressure in chickens appears to be age and gender dependent. In males is higher than in females [29]. Girard (1973) reported that one-day-old chicks have a systolic / diastolic blood pressure of 60.2 / 37.2 mm Hg and a heart rate of 156 beats/min. By 3–4 weeks these had risen to 114.6 / 72.9 mm Hg, 345 beats/min and at 5–6 weeks to 130.9 / 95.3 mm Hg and 376 beats/min [30]. The aorta acts as an elastic reservoir, storing blood transiently during systolic ejection, and providing an even flow to the periphery during diastole. Here the elastic recoil of the artery wall converts the pulsatile output of the heart into a smooth flow in the peripheral circulation [31].

Laplace’s law describes the tension in the walls of arteries. This geometric law applied to a tube explains that for a given internal fluid pressure, the wall tension will be proportional to the radius of the vessel [Wall tension = Transmural pressure × vessel radius / wall thickness] [32]. The application of this law to large arteries, that have comparable blood pressures, is that the larger the vessel radius, the greater the wall tension required to withstand a given internal fluid pressure. In the present study, the aortic wall thickness in birds of both LD and Ross lines increased with age as the vascular system adapted to higher blood pressures. When both lines had the same body weight, they had the same thickness of the aortic wall, but the luminal radius of aorta was 7.2% greater in LD birds than in the Ross birds. In accordance with Laplace’s law, when the transmural pressure is similar in both lines, the LD aorta has a greater wall tension than the Ross chickens. Consequently, the LD birds must have a stronger aortic wall to withstand the wall tension than that found in the Ross aorta. However, the mechanical properties of the arterial wall depend not only on the arterial wall thickness but also on the micro-architecture and composition of the arterial wall [33]. The vast majority of the aortic wall is made up of layers of smooth muscle embedded in elastin fibers, alternating with layers of collagen [31]. Elastin is a protein with rubber-like properties arranged as a network of highly extensible fibers in the elastic artery wall [24]. In the present study the proportion of the elastic fibers in the tunica media of the aorta decreased consistently with age in both lines confirming Ruiz-Feria et al findings [34]. Sans and Moragas (1993) suggested that the decrease in elastin concentration was, in part, due to the increase of other components such as collagen, while the total elastin content did not change [35]. Another study of the thoracic aorta of chickens demonstrated that the percentage of elastic fibers increased from the 1^st^ to the 30^th^ day of age and then decreased from this point on until the age of 36 months [36]. The elastic fibers in the tunica media are arranged in concentric fenestrated layers, the lamellae. Our findings show that the number of elastic lamellae in both chicken lines did not change with age. Similarly, Wagenseil and Mecham (2009) reported that the number of elastic lamellae does not change after birth in the aorta of vertebrates such as rats, rabbits and pigs [37]. Other studies have reported between 25 and 30 elastic lamellae in the aortic tunica media of adult chickens [38] and swans [39]. Each elastic lamella alternates with a physically connected concentric ring of smooth muscle cells to form lamellar units. A single lamellar unit has elastic properties allowing it to work within and to withstand a set range of mechanical load, that is best described by the parameter ‘tension’ [40]. There is a direct relationship between the tension and the number of elastic lamellae units, i.e. the number of lamellar units increases as arterial wall tension increases [41]. Faury (2001) suggested, that the tension per lamella is constant across species [40]. It has been estimated that each individual elastic lamella withstands in the range of 1–3 Nm^-1^ (Newton × Meter^-1^) tension in mammalian species [33]. Our results showed that the aorta of LD birds had a proportionally larger number of elastic lamellae and greater percentage of elastic fibers giving the aortic wall of LD chickens the ability to withstand greater wall tension.

## Conclusion

Our study indicates that the heart of both LD and Ross chicken lines has similar ventricular walls geometry. Furthermore, the blood capillary density was greater in LD’s heart than that in Ross’s heart. The micro-architecture of aorta, specifically the number of elastic lamellae and the percentage of elastic fibers are greater in LD chickens than in Ross chickens. Therefore, LD chickens have better aortic mechanical properties than those of Ross chickens, suggesting that the LD’ aorta has a greater ability to adapt to changes of blood pressure, than that of the Ross chickens.

## Supporting information

S1 Table Number of elastic lamellae in the aortic wall, lamellae number per 1mm aortic wall and percentage area occupied by elastic fiber bundles of both chicken lines. All versus day post hatching.
(DOCX)
