# Supplementary material for: Heart ventricular histology and microvasculature together with aortic histology and elastic lamellar structure: A comparison of a novel dual-purpose to a broiler chicken line
Source: PLoS One. 2019 Mar 21;14(3):e0214158. doi: 10.1371/journal.pone.0214158 (PMC6428391; doi:10.1371/journal.pone.0214158)
Supplement: S1 Table — All versus day post hatching. (DOCX) [file pone.0214158.s001.docx]

**S1 Table. Number of elastic lamellae in the aortic wall, lamellae number per 1mm aortic wall and percentage area occupied by elastic fiber bundles of both chicken lines. All versus day post hatching.**

| **Age (days)** | **Line (n)** | **Number of elastic lamellae** | **Lamellae number** | **Area occupied by elastic fibers %** |
| --- | --- | --- | --- | --- |
|  |  | **Mean ± SEM** | **Mean ± SEM** | **Mean ± SEM** |
| **1** | **Ross (6)** | 19.63 ± 0.53 | 41.77 ± 1.72 | 55 ± 0.005 |
|  | **LD (6)** | 19.17 ± 0.36 | 47.39 ± 4.66 | 55 ± 0.022 |
| **7** | **Ross (6)** | 19.88 ± 0.4 | 34.08 ± 2.69 | 52 ± 0.006 |
|  | **LD (6)** | 19.71 ± 0.24 | 34.20 ± 1.77 | 52 ± 0.011 |
| **14** | **Ross (6)** | 21.04 ± 0.39 | 26.52 ± 1.04 | 43 ± 0.013 |
|  | **LD (6)** | 20.25 ± 0.27 | 32.44 ± 0.93 | 42 ± 0.021 |
| **19** | **Ross (6)** | 21.38 ± 0.39 | 24.94 ± 1.23 | 38 ± 0.024 |
| **21** | **Ross (6)** | 21.32 ± 0.27 | 23.34 ± 0.85 | 45 ± 0.016 |
|  | **LD (6)** | 20.33 ± 0.52 | 29.91 ± 1.13 | 51 ± 0.013 |
| **25** | **Ross (6)** | 21.25 ± 0.65 | 22.76 ± 1.5 | 42 ± 0.01 |
| **28** | **Ross (6)** | 22.50 ± 0.57 | 22.89 ± 0.56 | 42 ± 0.025 |
|  | **LD (6)** | 21.96 ± 0.12 | 29.08 ± 1.6 | 44 ± 0.028 |
| **32** | **Ross (6)** | 22.42 ± 0.23 | 23.99 ± 1.16 | 31 ± 0.028 |
|  | **LD (6)** | 21.04 ± 0.25 | 28.84 ± 1.07 | 27 ± 0.012 |
| **35** | **Ross (6)** | 22.46 ± 0.23 | 22.61 ± 0.81 | 24 ± 0.016 |
|  | **LD (6)** | 20.96 ± 0.36 | 27.88 ± 0.82 | 36 ± 0.022 |
| **42** | **LD (6)** | 21.38 ± 0.31 | 24.31 ± 0.91 | 32 ± 0.014 |
| **49** | **LD (6)** | 20.08 ± 0.43 | 23.30 ± 0.75 | 37 ± 0.031 |
| **56** | **LD (6)** | 20.67 ± 0.35 | 22.38 ± 0.69 | 41 ± 0.027 |
| **63** | **LD (6)** | 21.21 ± 0.28 | 23.83 ± 0.81 | 37 ± 0.037 |

Area occupied by elastic fibers %: percentage of elastic fibers per field view of aortic wall; Lamellae number: number of lamellae per 1mm of aortic wall; LD: Lohmann Dual; Line: genetic line; n: animal number; Ross: Ross 308; SEM: standard error of the mean.
